# Thrombin-Derived Host-Defense Peptides Modulate Monocyte/Macrophage Inflammatory Responses to Gram-Negative Bacteria

**DOI:** 10.3389/fimmu.2017.00843

**Published:** 2017-07-21

**Authors:** Finja C. Hansen, Ann-Charlotte Strömdahl, Matthias Mörgelin, Artur Schmidtchen, Mariena J. A. van der Plas

**Affiliations:** ^1^Division of Dermatology and Venereology, Department of Clinical Sciences Lund, Lund University, Lund, Sweden; ^2^Division of Infection Medicine, Department of Clinical Sciences Lund, Lund University, Lund, Sweden; ^3^Dermatology and Venereology, Skåne University Hospital, Lund, Sweden; ^4^Dermatology, Lee Kong Chian School of Medicine, Nanyang Technological University, Singapore, Singapore

**Keywords:** host-defense peptides, Gram-negative bacteria, monocytes, macrophages, thrombin, phagocytosis, inflammation

## Abstract

Host-defense peptides play a fundamental role in the innate immune system by modulating inflammatory responses. Previously, it was shown that the thrombin derived host-defense peptide GKY25 inhibits LPS-induced responses of monocytes and macrophages *in vitro, ex vivo*, and *in vivo*. In this study, the effect of GKY25 on the interaction of monocytes/macrophages with Gram-negative bacteria was explored. Electron microscopy analysis showed that fibrin slough from non-healing wounds, colonized with *Staphylococcus aureus* and *Pseudomonas aeruginosa*, contains C-terminal thrombin epitopes associated with these bacteria extracellularly and in phagosomes of leukocytes. Live imaging of RAW 264.7 cell cultures showed binding of GKY25 to *Escherichia coli* BioParticles extracellularly, and colocalization intracellularly. Although peptide binding did not alter the rate of phagocytosis, GKY25 reduced NF-κB/AP-1 activation and subsequent cytokine release in response to both heat-killed and live bacteria. Notably, preincubation of RAW 264.7 cells with peptide did increase BioParticle uptake in a dose-dependent manner. Taken together, the thrombin-derived host-defense peptide GKY25 binds to bacteria extracellularly and colocalizes with bacteria intracellularly, thereby reducing pro-inflammatory responses.

## Introduction

Monocytes and monocyte-derived macrophages play an essential role during infection and wound healing. These phagocytic cells have various functions in host defense, such as engulfment of invading pathogens, release of pro-inflammatory cytokines and release of reactive oxygen species, to clear the infection at the site of injury (1, 2). In addition, macrophages remove apoptotic cells and promote cell proliferation of endothelial cells and fibroblasts through the release of growth factors and cytokines (3). Besides immune cells, host-defense peptides (HDPs) also play a fundamental role in the innate immune system. These peptides may exert multifunctional properties such as direct killing of bacteria, as well as modulating complement activation, coagulation, cytokine production, macrophage differentiation, reepithelialization, and other inflammatory responses (4–10). As a result, HDPs have attracted significant attention as potential novel anti-infectives (11, 12).

Previously, we showed that proteolytic degradation of human thrombin by neutrophil elastase generates thrombin-derived C-terminal peptides (TCPs) including, amongst other low molecular weight fragments, the 18 amino acid long peptide HVF18 (HVFRLKKWIQKVIDQFGE), which exerts host-defense activities (13, 14). Thus, although the primary role of thrombin during tissue damage and infection is to form a fibrin clot, it fulfills an additional role by the generation of multifunctional HDPs. Furthermore, we showed that the prototypic TCP GKY25 (GKYGFYTHVFRLKKWIQKVIDQFGE), like HVF18, reduces pro-inflammatory cytokine responses and modulates contact activation and tissue factor mediated clotting in response to LPS *in vitro*. It also increases survival of mice during LPS-induced shock by inhibiting cytokine release and decreasing fibrin deposition and leakage in the lungs (13, 14). Moreover, we found that GKY25 binds to LPS, thereby preventing LPS-induced toll-like receptor 4 (TLR4) dimerization on monocytes and macrophages, leading to inhibited downstream activation of transcription factors NF-κB and AP-1 and subsequent release of pro-inflammatory cytokines (15).

Whereas the effect of GKY25 on LPS-induced inflammatory responses is well established, its effect on bacteria and their interaction with immune cells is unclear. TCPs bind bacteria and exert antibacterial activity *in vitro* under standard buffer conditions (13), however mimicking physiological conditions by adding NaCl and plasma reduced this activity, while the presence of whole blood abrogated their bactericidal effects (16). In agreement, classical antimicrobial peptides, such as LL-37, likely exert little or no antimicrobial activity *in vivo*, while the immunomodulatory effects dominate at physiological concentrations (11, 17). In line with this, we recently reported that *Pseudomonas aeruginosa* mimics the formation of HDPs from thrombin in non-healing wounds, thereby enabling modulation and circumvention of host responses (16). Similar actions were found for *Staphylococcus aureus* (Saravanan et al., submitted), suggesting that formation of these peptides could be common for many M4 peptidase-producing bacteria.

Based upon the above findings that GKY25 inhibits LPS-induced inflammatory responses by monocytes and macrophages, and that TCPs bind to bacteria but do not exert direct antibactericidal effects under physiological conditions, we hypothesized that GKY25 may influence bacterial uptake and activation of monocytes and macrophages by Gram-negative bacteria.

Using electron microscopy, we show that C-terminal thrombin epitopes are found on bacterial surfaces inside phagosomes of leukocytes present in fibrin slough derived from non-healing, infected wounds. In agreement, binding of GKY25 to *Escherichia coli* BioParticles *in vitro* did not negatively affect phagocytosis. However, the peptide did not act as an opsonin either. Nevertheless, GKY25 reduced NF-κB/AP-1 activation and subsequent cytokine release in response to Gram-negative bacteria. Preincubation of RAW macrophages, but not monocyte-derived M1 or M2 macrophages, with GKY25 significantly increased phagocytosis. Taken together, the thrombin-derived HDP GKY25 reduces bacteria-induced activation of monocytes and macrophages, while preserving their import phagocytic function.

## Materials and Methods

### Peptides

The thrombin-derived peptides GKY25 (GKYGFYTHVFRLKKWIQKVIDQFGE), IVE25 (IVEGSDAEIGMSPWQVMLFRKSPQE), and HVF18 (HVFRLKKWIQKVIDQFGE) were synthesized by Biopeptide Co., Inc. (USA). The purity (>95%) of these peptides was confirmed by mass spectral analysis (MALDI-TOF Voyager).

### Microorganisms

*Escherichia coli* ATCC 25922 and *Pseudomonas aeruginosa* PAO1 were purchased from American Type Culture Collection (ATCC, USA). Bacteria from overnight cultures were grown to mid-logarithmic phase in Todd-Hewitt (TH) medium, centrifuged at 2,000 × *g* for 10 min and washed in 10 mM Tris buffer (pH 7.4). Next, *E. coli* was heat-inactivated for 20 min and *P. aeruginosa* for 30 min at 65°C. Killing was confirmed by plating the heat-inactivated suspensions on TH-agar plates.

### RAW Cell Cultures

RAW 264.7 cells (ATCC) and the derived RAW-Blue NF-κB/AP-1 reporter cells (InvivoGen, USA) were cultured in DMEM (HyClone, GE Healthcare Life Science, USA) supplemented with 10% (v/v) heat-inactivated FBS (FBSi; Invitrogen, USA) and 1% (v/v) antibiotic–antimycotic solution (AA; Invitrogen). In addition, 200 µg/ml of the selective antibiotic Zeocin (InvivoGen) was added to the RAW-Blue cells.

### Human Macrophages

Human peripheral blood mononuclear cells were isolated from buffy coats (obtained from Skåne University Hospital, Lund, Sweden) by Lymphoprep (ρ = 1.077 g/ml; Axis-Shield, Norway) density centrifugation at 700 × *g* for 20 min. Cells from the interphase were washed three times with phosphate-buffered saline (PBS) and monocytes were purified using anti-CD14-coated microbeads (Miltenyi Biotec, Germany) according to the manufacturer’s instruction (monocyte purity was >96%). To obtain M1 or M2 macrophages, cells were cultured for 6 days in RPMI-1640-GlutaMAX-I medium (Life Technologies, UK), 10% (v/v) FBSi, 1% (v/v) AA, and 5 ng/ml granulocyte macrophage colony-stimulating factor (GM-CSF; R&D Systems, UK) or 12.5 ng/ml macrophage colony-stimulating factor (M-CSF; R&D Systems) as described previously (18).

### NF-κB and AP-1 Activity Measurement

RAW-Blue reporter cells (4 × 10^5^ cells/ml) in DMEM supplemented with 10% FBSi were seeded in 96-wells plates and incubated overnight to allow adherence. Next, the cells were incubated with 4 × 10^5^ CFU/ml of heat-inactivated *E. coli* or 4 × 10^6^ CFU/ml of heat-inactivated *P. aeruginosa* in the presence or absence of peptides using various conditions. After 20 h, NF-κB/AP-1 activation was determined by the release of secreted embryonic alkaline phosphatase (SEAP) into the culture supernatants. For this purpose, 20 µl supernatant was mixed with 180 µl SEAP detection reagent (QUANTI-Blue, InvivoGen), incubated for 1.5 h at 37°C, and the absorbance was measured at 600 nm.

### Measurement of Cytokine Levels

RAW 264.7 cells (4 × 10^5^ cells/ml) in DMEM with 10% FBSi were seeded in 96-wells plates and incubated overnight to allow adherence. Cells were stimulated with heat-inactivated *E. coli* (4 × 10^5^ CFU/ml) or *P. aeruginosa* (4 × 10^6^ CFU/ml), as described previously for 20 h in the presence or absence of GKY25 and IVE25. Cytokines in supernatants of RAW 264.7 cell cultures were determined using the CBA Mouse Inflammation Kit (BD Biosciences, USA) according to the manufacturer’s protocol.

To determine cytokine release by human cells, fresh venous blood taken from healthy donors, collected in the presence of the anticoagulant lepirudin (50 µg/ml) was mixed with 3 parts RPMI-1640-GlutaMAX-I medium. Next, 1 ml of this mixture was stimulated in 24-wells plates with *E. coli* (4 × 10^3^ CFU/ml) or *P. aeruginosa* (4 × 10^4^ CFU/ml) in the presence or absence of 10 µM GKY25. After 6 h of incubation at 37°C and 5% CO_2_, plates were centrifuged for 5 min at 200 × *g* without break and plasma was collected for cytokine analysis. The levels of IL-6, IL-8, IL-12p40 and TNF-α were determined using Human CytoSets (Invitrogen), whereas the Human Cytokine Array panel A (R&D Systems) was used to analyze a panel of 36 cytokines and chemokines according to the manufacturer’s instructions.

### Phagocytosis Assay

RAW 264.7, M1, or M2 macrophages (8 × 10^5^ cells/ml) were seeded in 96-wells plates and incubated overnight to allow adherence. The following day, cell medium was refreshed, and the cells were preincubated with different concentrations of GKY25. After 3 h of incubation, the cell medium was removed and fluorescent pHrodo Green *E. coli* BioParticles (Life Technologies), suspended in Hank’s balanced salt solution (HBSS) according to the manufacturer’s instructions, were added to the cells. In a separate set of experiments, *E. coli* BioParticles were preincubated with GKY25 in the presence or absence of AB serum for 1 h prior to addition to naive cells. After 1.5 h incubation of cells and particles at 37°C, the mean fluorescence of phagocytosed particles was measured using a VICTOR3 Multilable Plate counter spectrofluorometer (PerkinElmer, USA) at excitation/emission wavelengths of 485/535 nm. Wells containing only fluorescent *E. coli* BioParticles were used as a negative control, whereas cells incubated with fluorescent *E. coli* BioParticles in the absence of GKY25 were used as a positive control. Phagocytosis is expressed as the percentage of fluorescence as compared to the positive (100%) and negative (0%) controls. Each experiment was carried out in quadruplicate, and the results were calculated from three to six independent experiments.

### Cell Viability Test

The viability of RAW-Blue and RAW 264.7 cells was examined by a 3-(4,5-dimethylthiazol-2-yl)-2,5-diphenyltetrazolium bromide (MTT, Sigma-Aldrich, USA) reduction assay. In brief, cells were incubated with 10% (v/v) of a 5 mg/ml MTT solution for 1 h at 37°C, washed with PBS and 100 µl dimethyl sulfoxide was added followed by measuring of the absorbance at a wavelength of 550 nm.

### Western Blot

Samples were diluted in reducing Tricine SDS Sample Buffer (Life Technologies) and denatured at 95°C for 7 min. Next, samples were separated by electrophoresis on a 10–20% Tris-Tricine gel (Life Technologies) using 1× Tris–Tricine SDS running buffer (Life Technologies) for 100 min at 100 V. Proteins and peptides were transferred to PVDF membranes at 25 V for 10 min using a Trans Blot Turbo system (BioRad, USA). Subsequently, membranes were blocked with 3% non-fat dry milk in PBS containing 0.05% Tween-20 (PBST) for 30 min at RT. TCPs were detected using polyclonal rabbit antibodies against the thrombin C-terminal epitope VFRLKKWIQKVIDQFGE (VFR17; Innovagen AB, Sweden) for 1 h at RT, followed by three times washing with PBST and incubation with swine anti-rabbit immunoglobulin-HRP conjugated antibodies (1:1,000; Dako, Denmark) for 1 h at RT. Membranes were washed thrice with PBST, and TCPs were visualized after 3 min incubation with SuperSignal West Pico Chemiluminescent Substrate (Thermo Scientific, Denmark) using the Chemi Doc MP imaging system (BioRad).

### Electron Microscopy

Fibrin slough from a patient with a chronic venous ulcer was fixed with 2.5% glutaraldehyde in cacodylate buffer and processed as previously described (13). For immunostaining of TCPs on bacteria, samples were blocked with 5% (v/v) goat serum in incubation buffer [0.2% bovine serum albumin (BSA) in PBS, pH 7.6] for 15 min at RT followed by overnight incubation with 1 µg/ml of VFR17 at 4°C. Next, samples were washed and incubated with 1 µg/ml EM goat anti-Rabbit IgG 10 nm Au (BBI, UK), diluted in incubation buffer, for 2 h at 4°C. After additional washing and post fixation with 2% glutaraldehyde, samples were examined using a FEI/Philips CM 100 transmission electron microscope (operated at 80 kV accelerating voltage) connected to an Olympus Veleta camera (Japan).

### Confocal Microscopy

RAW 264.7 cells (2 × 10^5^ cells/ml) were seeded on round glass cover slips (Menzel-Gläser, Thermo Scientific) and incubated overnight to allow adherence. pHrodo Green *E. coli* BioParticles were suspended as described above and incubated with 2 µM GKY25 or 20 µM HVF18 for 1 h at 37°C. Next, preincubated *E. coli* BioParticles were added to RAW 264.7 for 1.5 h at 37°C to allow phagocytosis. Afterward, cells were washed with PBS, fixed with 2% formaldehyde for 30 min at 4°C, washed again and permeabilized with 0.5% (v/v) Triton for 2 min at RT. After washing, samples were blocked with 5% (w/v) BSA in PBS, incubated with VFR17 for 30 min at 37°C, washed again with PBS and incubated with secondary goat anti-rabbit Alexa Fluor 568 antibody (1:1,000; Invitrogen) for 30 min at 37°C. Subsequently, samples were washed and mounted with ProLong Gold with DAPI (Life Technologies) to stain the nuclei. Mounted samples were examined using an LSM 700 laser-scanning confocal microscope (Zeiss, Germany) with λ_ex_ = 405 nm for DAPI, λ_ex_ = 488 nm for *E. coli* BioParticles, and λ_ex_ = 568 nm for GKY25 or HVF18. A C-Apochromat 63×/1.20 W Korr M27 glycerol immersion objective was used. Images were collected and processed with Zen 2012 software and analyzed using the ImageJ software (version 1.49q).

### Live Cell Imaging

RAW 264.7 cells (2 × 10^5^ cells/ml) were cultured in glass bottom cell culture dishes (VWR, Sweden) overnight to allow adherence. The next day, cell medium was changed to DMEM supplemented with 10% (v/v) AA (no FBSi), and *E. coli* BioParticles (50 µg/ml), suspended in HBSS medium, were added together with 2 µM TAMRA-labeled (T-) GKY25 to the RAW 264.7 cells. The culture dishes were placed in an environmental chamber (37°C and 5% CO_2_), and images were immediately recorded every third minute for 2 h, from three different areas, with the Nikon A1+ confocal on a TiE inverted microscope (Nikon, Japan) with GaAsO detectors. Images were collected using a 60×/1.4 plan Apo λs lens. The λ_ex_ = 488 nm was selected for green-labeled *E. coli* BioParticles and λ_ex_ = 561 nm for T-GKY25. Images were captured and processed using the NIS-Elements software (Nikon).

### Statistical Analysis

Values are shown as means ± SEM of “*n*” independent experiments. Evaluation of the differences between control and test samples was done in GraphPad Prism version 6.0 using a one-way ANOVA with a Dunnett’s multiple comparisons test or a paired *t*-test. A *p* value of <0.05 was considered significant. The heat map was created in R (19) with the R package ggplot2 (20).

## Results

### Thrombin C-Terminal Peptides Are Binding to Bacteria in Chronic Wounds

**Figure 1:**
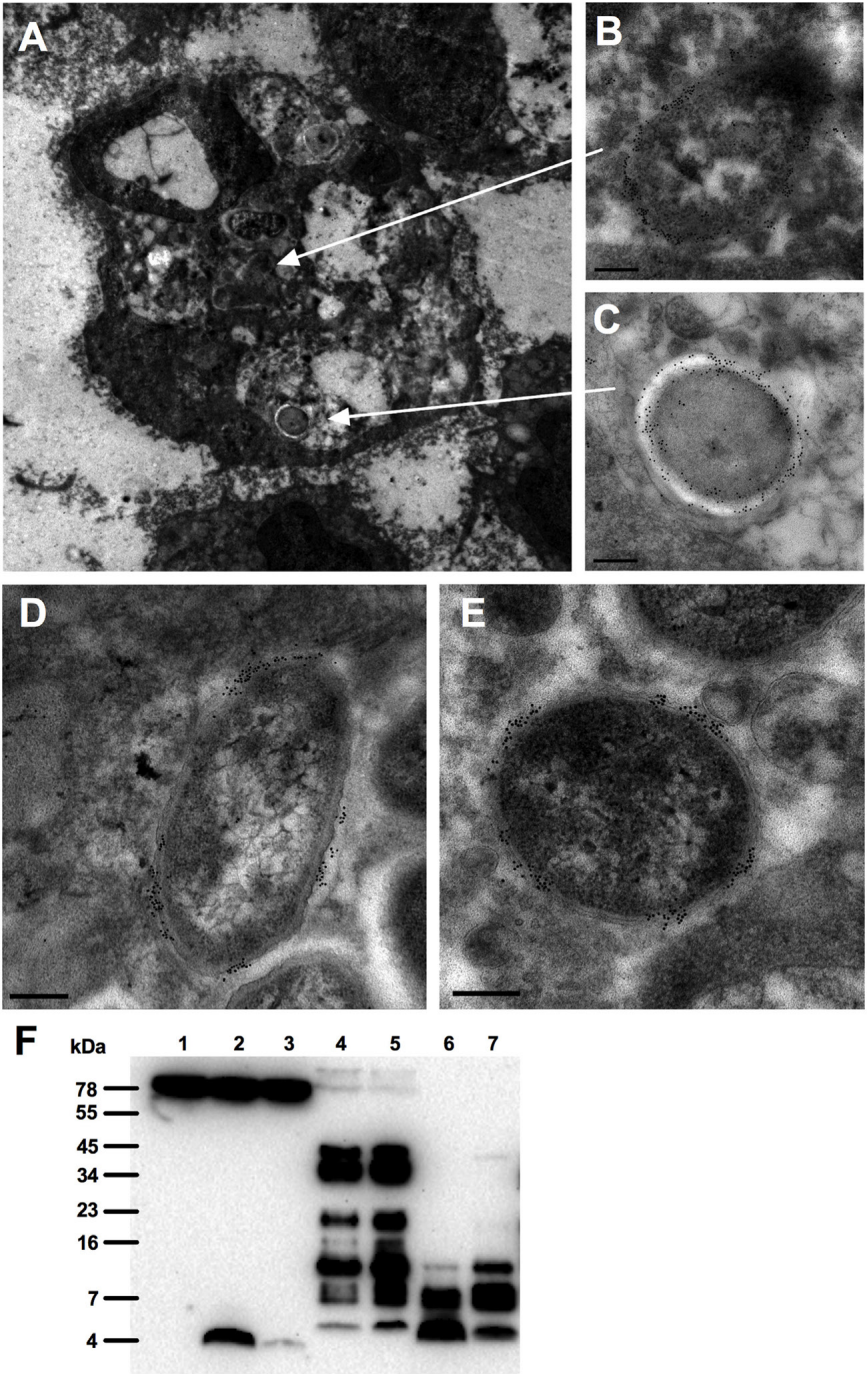
Interactions of thrombin-derived C-terminal peptides with bacteria in wounds. Thrombin-derived C-terminal peptides are visualized in fibrin slough by gold labeling (indicated as black dots) using electron microscopy. **(A)** Overview of a polymorphonuclear leukocyte in fibrin slough from a patient with a non-healing venous ulcer colonized with *Pseudomonas aeruginosa* and *Staphylococcus aureus*, showing the binding of thrombin-derived C-terminal peptide (TCP) epitopes to a phagocytosed bacillus **(B)** and a coccus **(C)**. In addition, TCPs are bound to a bacillus **(D)** and a coccus **(E)** that are present extracellularly in the fibrin slough. Scale bar, 0.2 µm. **(F)** Western blot analysis detected the presence of TCPs in acute (lanes 4–5) and chronic (lanes 6–7) wound fluids. Human plasma is shown in lane 1, human plasma with GKY25 in lane 2 or with HVF18 in lane 3.

As chronic, non-healing ulcers are commonly colonized or infected by bacteria such as *S. aureus* and *P. aeruginosa* (21, 22), we explored whether TCPs are associated with bacteria in these wounds. For this purpose, fibrin slough was collected from a wound containing *P. aeruginosa* (rod shaped) and *S. aureus* (cocci) and analyzed using electron microscopy. Figure 1A illustrates the presence of both rods and cocci in a phagocytic cell located in the fibrin slough; TCPs were found to be present on both types of bacteria (Figures 1B,C). Furthermore, TCPs also interacted extracellularly, in the fibrin slough, with these bacteria (Figures 1D,E). The presence of TCPs in acute wound fluids and chronic wound fluids was confirmed by western blot analysis (Figure 1F). TCPs of approximately 4 kDa are comparable with both the TCP HVF18, a peptide cleaved by neutrophil elastase (13), and the three amino acid larger TCP FYT21, cleaved by *Pseudomonas* elastase (16), as well as the prototypic TCP GKY25.

### GKY25 Binds to *E. coli* BioParticles in the Extracellular Environment

To investigate if TCPs are interacting with bacteria intracellularly or extracellularly, *E. coli* BioParticles were used and added together with TAMRA-labeled (T)-GKY25 to RAW 264.7, and the uptake was recorded for 2 h by using live cell imaging. The videos (Videos S1 and S2 in Supplementary Material) demonstrate direct binding of T-GKY25 to *E. coli* BioParticles extracellularly. Phagocytosis by RAW 264.7 cells of *E. coli* BioParticles with bound T-GKY25 was observed 10 min after the start of the experiments. In addition, RAW 264.7 cells internalized small aggregates of *E. coli* BioParticles with bound T-GKY25 as well as peptide alone. Control samples without peptides show uptake of *E. coli* BioParticles in RAW 264.7 cells, whereas control samples without *E. coli* particles show the presence of GKY25 and HVF18 in the cytoplasma of the RAW 264.7 cells (Figure S1 in Supplementary Material). Of note, the control peptide IVE25 did not bind to *E. coli* BioParticles or RAW 264.7 cells (data not shown).

**Figure 2:**
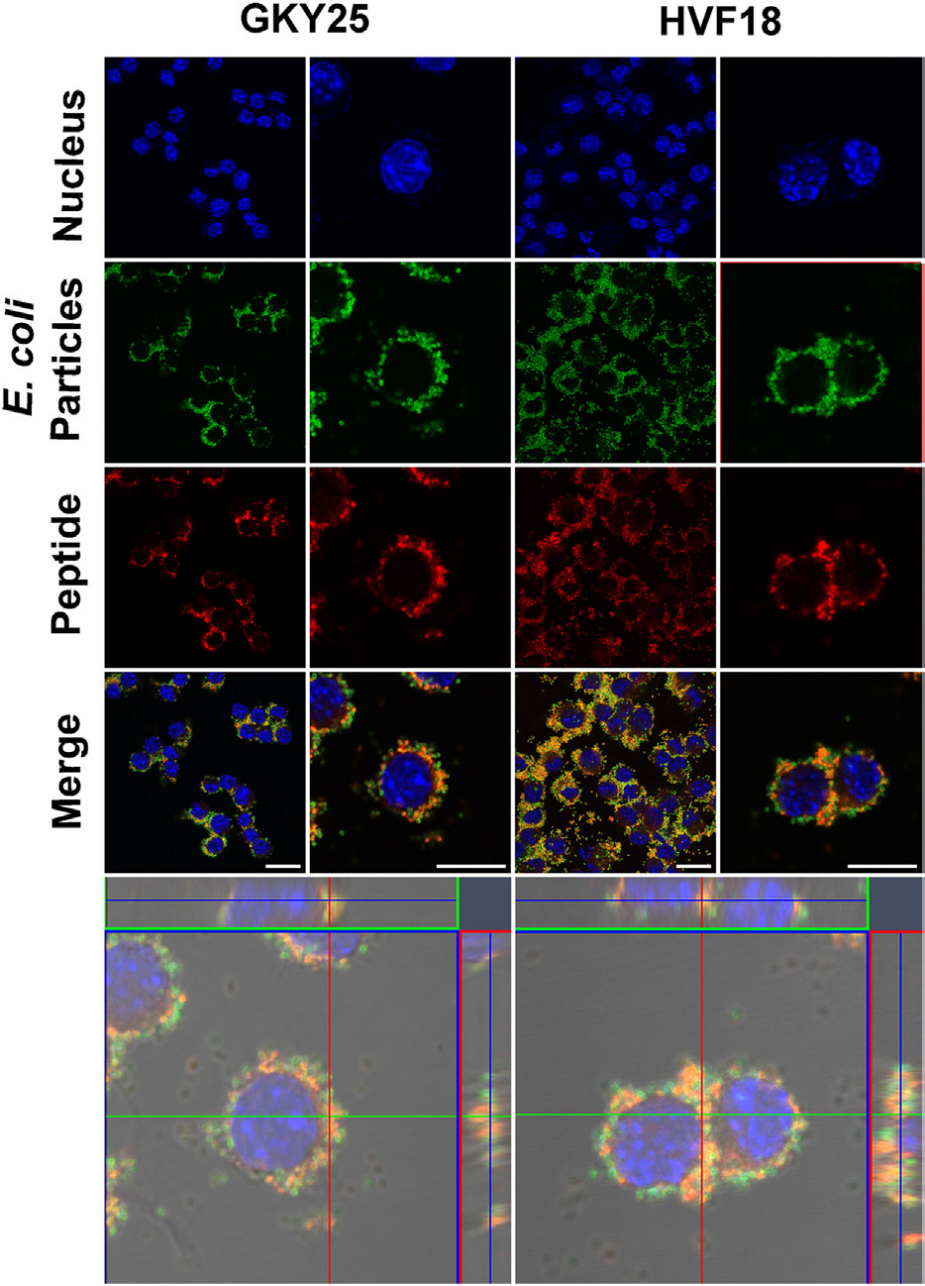
Phagocytosis of *Escherichia coli* BioParticles with bound thrombin-derived C-terminal peptides (TCPs) by RAW 264.7 cells. *E. coli* BioParticles were incubated with GKY25 (2 µM) or HVF18 (20 µM) for 1 h before addition to RAW 264.7 cells. Next, cells were fixed, stained and analyzed *via* confocal microscopy using Zeiss LSM software. Nucleus DNA was stained with DAPI (blue), *E. coli* BioParticles are green, and TCPs were labeled with VFR17 antibody and Alexa 568 (red). The left panel shows an overview, and the right panel shows a higher magnification of the cells. The lower panel displays a z-stack of the RAW 264.7 cells with a top view looking down to the cell (square) and a side view through the cell (right and above). One representative image out of three independent experiments is shown. Scale bar, 10 µm.

Furthermore, to confirm the interaction of TCPs with *E. coli* BioParticles before entering the cells, *E. coli* BioParticles were preincubated with T-GKY25 or T-HVF18 for 1 h followed by the addition to RAW 264.7 cells. The results show internalization of *E. coli* BioParticles colocalized with GKY25 or HVF18 in RAW 264.7 cells (Figure 2).

### GKY25 Modulates Phagocytosis

**Figure 3:**
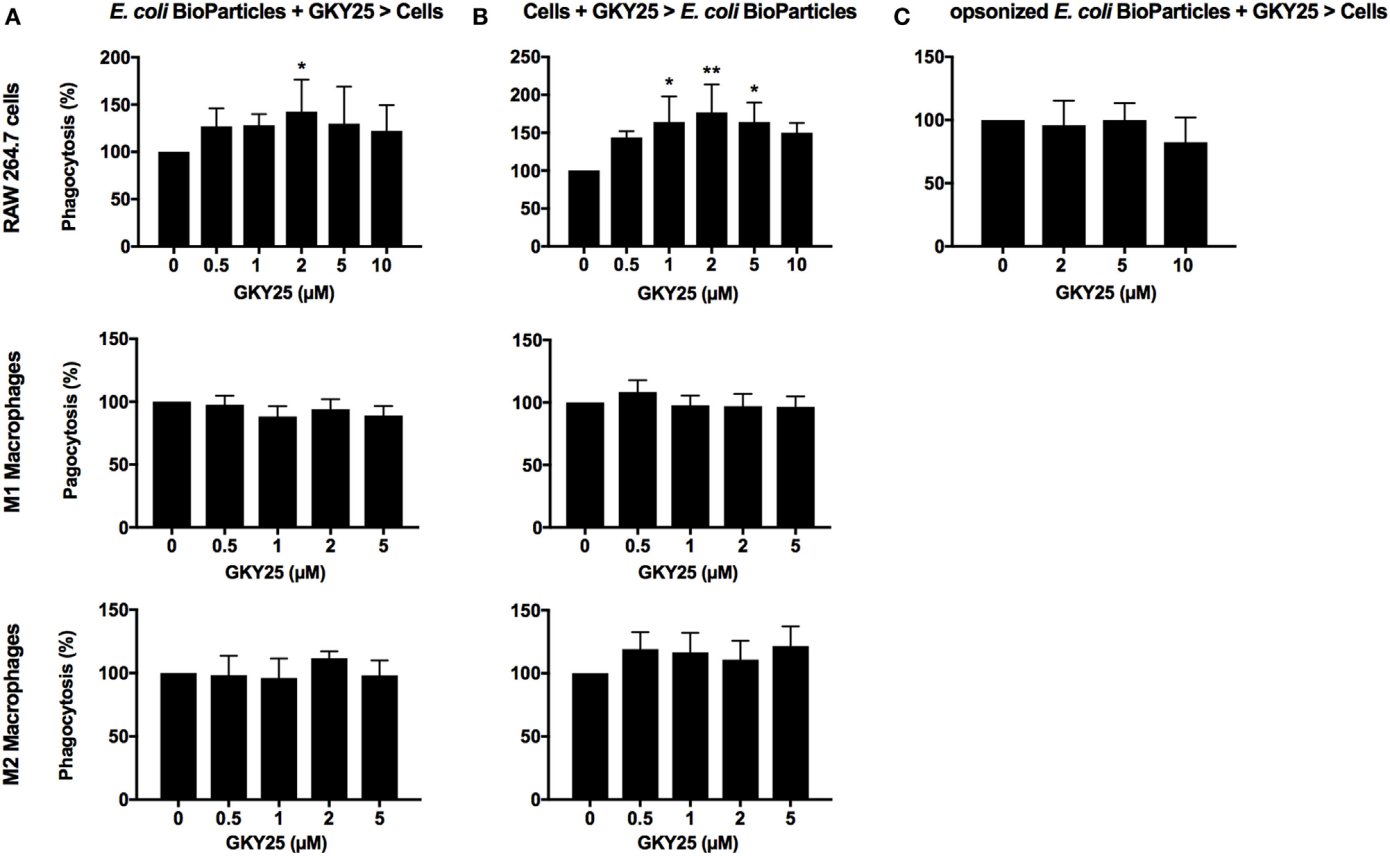
Effects of GKY25 on phagocytosis of *Escherichia coli* BioParticles by macrophages. RAW 264.7, M1, and M2 macrophages (8 × 10^5^ cells/ml) were seeded in 96-wells plates and incubated overnight to allow adherence. **(A)** *E. coli* BioParticles were preincubated with a range of GKY25 for 1 h before addition to the cells (*E. coli* BioParticles + GKY25 > cells). **(B)** RAW 264.7, M1, and M2 macrophages were incubated with GKY25 for 3 h (Cells + GKY25 > *E. coli* BioParticles) followed by addition of the *E. coli* BioParticles. Results are means ± SEM of *n* = 3–6 (RAW 264.7), *n* = 6 (M1 macrophages), and *n* = 3–4 (M2 macrophages) experiments. Values are significantly (*p < 0.05 and **p < 0.005) different from the controls as analyzed using a one-way ANOVA with a Dunnett’s multiple comparisons test. **(C)** *E. coli* BioParticles were opsonized with 10% AB serum in the presence of GKY25 for 1 h at 37°C before addition to the cells (opsonized *E. coli* BioParticles + GKY25 > cells). Results are means ± SEM of *n* = 3 experiments. In all experiments, RAW 264.7 cells were incubated with *E. coli* BioParticles for 1.5 h at 37°C. The fluorescence of phagocytosed *E. coli* BioParticles was measured at 485/535 nm, and values were calculated as described in the Section “Materials and Methods.”

As TCPs are interacting with bacteria extracellularly, we explored whether TCPs affect phagocytosis. To investigate if GKY25 has opsonizing effects, *E. coli* BioParticles were preincubated for 1 h with a range of GKY25, before addition to the cells. We found that the uptake of the particles by RAW 264.7 cells was slightly affected with 2 µM of GKY25 (Figure 3A) but not with any of the other tested concentrations or when using monocyte-derived M1 or M2 macrophages. Interestingly, 3 h preincubation of RAW 264.7 cells with GKY25 significantly increased phagocytosis of *E. coli* BioParticles (Figure 3B) in a dose-dependent fashion. Incubation times up to 24 h of cells with peptide did not further affect the phagocytic activity of RAW 264.7 cells (data not shown). Of note, phagocytosis by monocyte-derived M1 and M2 macrophages was not significantly affected by the peptide. Furthermore, no difference in phagocytosis of BioParticles was observed when serum-opsonized in the presence of GKY25 (Figure 3C). Finally, preincubation of *E. coli* BioParticles with 20 µM HVF18 did not affect phagocytosis by RAW 264.7 cells (data not shown).

### GKY25 Reduces Bacteria-Induced NF-κB/AP-1 Activation

**Figure 4:**
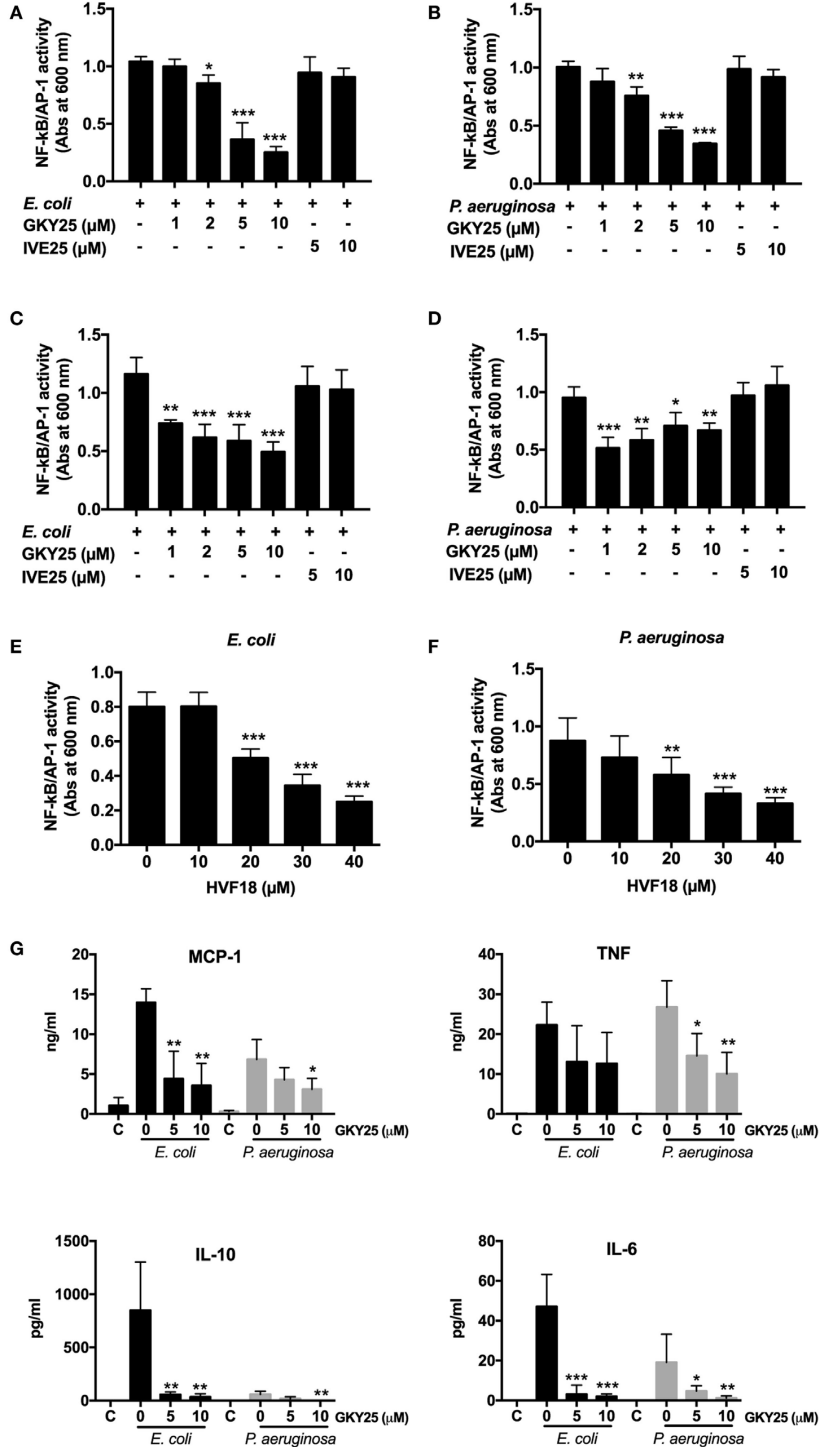
GKY25 reduces NF-κB/AP-1 activation after stimulation with heat-killed bacteria. **(A,B)** RAW-Blue cells were stimulated with heat-killed *Escherichia coli* (4 × 10^5^ CFU/ml) or *Pseudomonas aeruginosa* (4 × 10^6^ CFU/ml) in the presence of the indicated concentrations of GKY25 and IVE25. **(C,D)** Heat-killed *E. coli* (4 × 10^5^ CFU/ml) or *P. aeruginosa* (4 × 10^6^ CFU/ml) were preincubated for 2 h at 37°C with GKY25, washed and added to RAW-Blue cells. **(E,F)** RAW-Blue cells were stimulated with heat-killed *E. coli* (4 × 10^5^ CFU/ml) or *P. aeruginosa* (4 × 10^6^ CFU/ml) in the presence of HVF18. After 20 h of incubation, NF-κB/AP-1 activation was measured in the cell supernatants by using QUANTI-Blue reagent, and absorbance was measured at 600 nm. **(G)** RAW 264.7 cells were stimulated with heat-killed *E. coli* (4 × 10^5^ CFU/ml) or *P. aeruginosa* (4 × 10^6^ CFU/ml) in the presence of the indicated concentrations of GKY25 for 20 h. Cytokine release was determined in the cell supernatants. Non-stimulated cells are indicated with C. Results are means ± SEM of *n* = 3–5 experiments. Values are significantly (*p < 0.05, **p < 0.005, and ***p < 0.0005) different from the controls as analyzed using a one-way ANOVA with a Dunnett’s multiple comparisons test.

As GKY25 is preserving the bacterial uptake by RAW macrophages, we investigated whether the peptide would modulate bacteria-induced cell activation. The results showed that GKY25 dose-dependently inhibited NF-κB/AP-1 activation by RAW-Blue cells in response to heat-killed *E. coli* and *P. aeruginosa* (Figures 4A,B).

Preincubation of heat-killed bacteria with peptide for 2 h, followed by a washing step before addition to RAW-Blue cells, resulted in inhibited NF-κB/AP-1 activation as well, indicating that binding of GKY25 to heat-killed bacteria adequately inhibits cell activation (Figures 4C,D). The endogenous HVF18 showed similar effects as GKY25. However, to get a significant reduction in NF-κB/AP-1 activation, 20 µM of peptide was needed (Figures 4E,F).

In line with these results, the presence of GKY25 reduced the release of the cytokines IL-6, IL-10, MCP-1, and TNF by RAW 264.7 cells stimulated for 20 h with heat-killed *E. coli* and *P. aeruginosa* (Figure 4G); the release of IFN-γ and IL-12p70 was below detection limit (2 pg/ml). Interestingly, stimulation of the cells was much weaker in the presence of *P. aeruginosa* as compared to *E. coli*, except for the release of TNF, even though 10 times less CFU were used for the latter species. IVE25, derived from the N-terminal part of thrombin, was used as a control peptide and did not reduce NF-κB/AP-1 activation (Figures 4A–D) or the release of cytokines (data not shown). Of note, the used concentrations of bacteria and peptides did not significantly influence the viability of the cells (Figure S2 in Supplementary Material).

### GKY25 Affects Cytokine and Chemokine Secretion after Stimulation of Whole Blood with Live Bacteria

**Figure 5:**
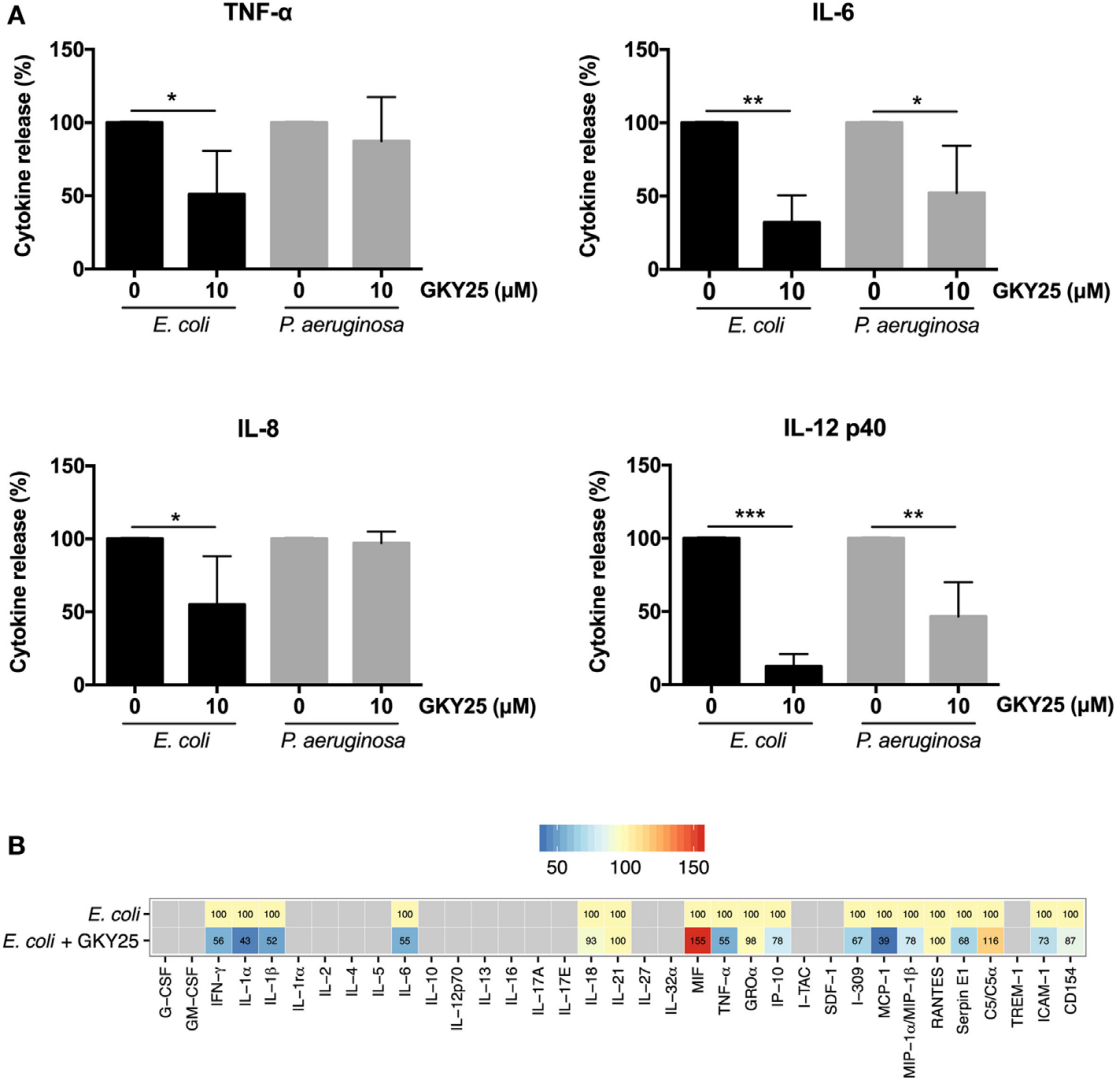
GKY25 reduces cytokine and chemokine release in response to live bacteria. **(A,B)** The release of cytokines in plasma was measured after human blood was incubated for 6 h with *Escherichia coli* or *Pseudomonas aeruginosa* in the presence or absence of 10 µM GKY25 (means ± SEM; *n* = 4; *p < 0.05, **p < 0.002, ***p < 0.0001 paired *t*-test). Gray quadrates in the heat map indicate not detected. The values demonstrate the median of five independent experiments, except for MIF and MCP-1 (four experiments) and I-309 (three experiments).

To investigate whether GKY25 can inhibit pro-inflammatory responses in the presence of live bacteria, whole blood was stimulated for 6 h with *E. coli* or *P. aeruginosa* together with 10 µM GKY25, followed by cytokine analysis of the cell-free supernatants. The results showed significant decreases in the release of TNF-α from 6.6 (range 3.1–10.9) to 2.9 (0–5.7) ng/ml, IL-6 from 17.1 (9.1–24.8) to 5.0 (0–10.8) ng/ml, IL-8 from 6.1 (3.8–9.3) to 2.8 (0.4–4.4) ng/ml, and IL-12p40 from 1.6 (0.7–2.9) to 0.2 (0.06–0.5) ng/ml by GKY25 in response to *E. coli* stimulation (Figure 5A). Furthermore, GKY25 significantly decreased the release of IL-6 from 2 (0.4–3.7) to 0.6 (0.3–1.2) ng/ml and IL-12 from 0.2 (0.04–0.6) to 0.1 (0.3–0.4) ng/ml after *P. aeruginosa* stimulation, whereas TNF-α (0.18 ng/ml) and IL-8 (1.2 ng/ml) were not significantly affected. More extensive cytokine and chemokine analysis of whole blood stimulated with *E. coli* using profiler arrays showed that GKY25 (10 µM) also reduced the cytokines IFN-γ, IL-1α, and IL-1β, and the chemokines IP-10, I-309, MCP-1, and MIP-1α/MIP-1β, as well as serpin E1, ICAM-1, and CD154 (Figure 5B). GKY25 (10 µM) itself did not induce increased cytokine or chemokine release as compared to unstimulated cells (data not shown).

## Discussion

The thrombin-derived HDP GKY25 exerts multiple immunomodulatory effects in response to LPS including inhibition of coagulation and cytokine release *in vitro* and *in vivo* (14, 15). We previously demonstrated that GKY25 binds to both LPS and cell membranes, thereby inhibiting TLR4 dimerization and subsequent downstream activation of transcription factors NF-κB and AP-1, as well as MyD88-independent pathways (15). Mechanistically, interactions with LPS, both in aqueous solution and in lipid membranes, were investigated for GKY25, using ellipsometry, dual polarization interferometry, fluorescence spectroscopy, circular dichroism, dynamic light scattering, and *z*-potential measurements. It was concluded that the anti-inflammatory effect is mediated by a combination of LPS/lipid A binding and facilitated by helix formation of GKY25 in the presence of the agonist (23). Besides affecting LPS-induced responses, GKY25 was also able to inhibit monocyte/macrophage responses to peptidoglycan, LTA, zymosan, and ODN1826 (14, 15), indicating that the inhibitory activity is not restricted to a specific TLR. Therefore, it seems plausible that cationic TCPs, such as GKY25, show similar electrostatic and/or hydrophobic interactions with these negatively charged agonists, the negatively charged cell membranes of bacteria and immune cells (13, 16) as well as anionic liposomes (23). However, the exact mode of action needs to be demonstrated in future mechanistic studies.

Whereas TCP-induced modulation of responses to pathogen associated molecular patterns is confirmed, the effect of these peptides on the interaction of whole bacteria and the immune system is unclear. In the present work, we show that binding of GKY25 to Gram-negative bacteria results in reduced activation of monocytes and macrophages while preserving their important phagocytic function. This conclusion is based on the following observations. First of all, live imaging of RAW 264.7 cell cultures showed binding of GKY25 to *E. coli* BioParticles extracellularly, and colocalization intracellularly. In agreement, electron microscopy analysis of fibrin slough from a patient with an infected non-healing leg ulcer showed rod-shaped and coccoid bacteria inside a leukocyte with thrombin-derived C-terminal epitopes bound to their surface. *In vitro* phagocytosis assays indicated that preincubation of 2 µM of GKY25 with *E. coli* BioParticles significantly increased phagocytosis by RAW 264.7 macrophages. However, the other tested concentrations only showed a trend, whereas GKY25 did not affect phagocytosis by M1 and M2 macrophages at all, making the physiological relevance of this finding ambiguous. Contrastingly, preincubation of RAW 264.7 cells with this peptide dose-dependently did increase phagocytosis. Clearly, it would be interesting to further investigate the exact mechanisms responsible for this increased bacterial uptake, but this is beyond the scope of the present study.

Although GKY25 does not exert a clear opsonizing effect, it did reduce NF-κB/AP-1 activation in response to heat-killed bacteria by monocytes and macrophages and the release of various pro-inflammatory cytokines after stimulation with live bacteria in whole blood studies. Thus, these results show that the previous reported inhibition of cell responses to purified bacterial products is applicable to whole bacteria as well. In addition, our previous finding that scavenging of LPS also takes place intracellularly in endosomes (15) further explains our current observation that cell activation is suppressed even when bacteria are taken up by the cells.

Of particular clinical relevance, and in agreement with our previous study (16), is the finding that TCPs are present in both acute and chronic wounds. After wounding, thrombin binds to fibrin with high affinity and subsequent proteolysis leads to release of thrombin fragments. Interestingly, in acute wound fluids we observed thrombin itself and a range of TCPs of different sizes, whereas in chronic wounds only smaller TCPs were present. In agreement, lack of intact thrombin in non-healing wounds has been reported previously (24) and is likely due to the high proteolytic environment in these wounds. Furthermore, we recently reported that *P. aeruginosa* elastase cleaves thrombin, leading to the formation of a 21 amino acid TCP (designated FYT21) that dampens inflammation, thereby mimicking the endogenous formation of thrombin-derived HDPs by neutrophil-derived elastase (16), to circumvent host responses. Evidently, the endogenous formation of these peptides, together with the bacterial hijacking of this system, implies a role of such thrombin peptides in modulating inflammation during wounding and infection. Although the concentration of TCPs in these wounds are unknown, given that the physiological concentration of human prothrombin in plasma is ~1.4 μM (25), and the fact that large amounts of fibrin and bound thrombin are deposited in wounds, we consider the concentrations of GKY25 used in our studies of physiological relevance. However, it should be noted that the endogenous formed TCP HVF18 significantly reduced NF-κB/AP-1 activation in RAW 264.7 cells in response to bacterial stimulation only when using 10 times higher concentrations than used for the seven amino acids larger GKY25. This may be explained by our earlier observation that an internal fragment of TCPs of 12 amino acids (VFRLKKWIQKVI) bound LPS but showed no anti-endotoxic effects upon macrophage stimulation (23, 26), indicating length-dependent interactions influenced by helix formation of these peptides. Current investigations address the exact structures of various TCPs and their physiological roles and concentrations during wounding and infection.

Whereas HDPs were mainly regarded as a possible source for the development of alternatives to antibiotics at first, recently the perspective on HDPs as therapeutics has changed, focusing more and more on their potential as immune modulators (27, 28), as their immunomodulatory effects dominate at physiological concentrations (11, 17). From a clinical and therapeutic viewpoint, thrombin-derived peptides may have potential to prevent detrimental inflammation, and exploration of their modes of action is therefore clearly motivated. Previous studies showed that the prototypic GKY25 reduces coagulation and pro-inflammatory cell responses, and increases survival of mice in experimental models of endotoxin shock and *P. aeruginosa* sepsis (13, 14). In the latter model, treatment with GKY25 showed only marginal effects on bacterial levels, whereas the reduction of pro-inflammatory cytokine release was evident (14). In agreement, our previous finding that TCPs do not exert direct antibactericidal effects in the presence of whole blood together with our current findings, showing that binding of GKY25 or HVF18 to bacteria does not influence their phagocytosis but suppresses monocyte/macrophage activation, clearly implies that immunomodulation is the dominating effect of TCPs *in vivo*. Therefore, in some circumstances, such as infected wounds or sepsis, these peptides should be used together with antibiotics to address both infection and inflammation. Notably, current unpublished research shows a potential role for TCPs as stand-alone treatments preventing bacterial infections in acute wounds.

Taken together, the actions of GKY25 and HVF18 observed in the present study, together with previous findings on the working mechanisms of various TCPs, show that thrombin-derived peptides play a physiological role during infection and inflammation, and may have therapeutic potential by modulating multiple interactions involving bacteria, endotoxins, and inflammatory cells responses.

## Ethics Statement

This study was carried out in accordance with the recommendations of the Ethics Committee at Lund University, Lund, Sweden with written informed consent from all subjects. All subjects gave written informed consent in accordance with the Declaration of Helsinki. The protocols for the use of human blood (permit no. 657-2008) and human wound materials (LU 708-01, LU 509-01) were approved by the Ethics Committee at Lund University.

## Author Contributions

FH, AS, and MP conceived and designed the experiments. FH and A-CS performed the *in vitro* cell studies. MM did the electron microscopy studies. FH analyzed the data and prepared the figures. FH and MP wrote the paper. All the authors discussed the results and commented on the manuscript.

## Conflict of Interest Statement

AS is a founder of in2cure AB, a company developing anti-inflammatory peptides of thrombin for therapeutic applications (patents US8735353B2, EP2480567B1, EP1987056B1, and US8076286B2). The other authors declare no competing financial interests.

## Supplementary Material

Video S1 Overview of the phagocytosis of *E.coli* BioParticles in the presence of T-GKY25.

Video S2 3D projection of the phagocytosis of *E.coli* BioParticles in the presence of T-GKY25.
