# Supplementary material for: Thrombin-Derived Host-Defense Peptides Modulate Monocyte/Macrophage Inflammatory Responses to Gram-Negative Bacteria
Source: Front Immunol. 2017 Jul 21;8:843. doi: 10.3389/fimmu.2017.00843 (PMC5519531; doi:10.3389/fimmu.2017.00843)
Supplement: Supplementary file 3 [file data_sheet_1.pdf]

Supplementary Material

Thrombin-Derived Host Defense Peptides modulate Monocyte/Macrophage Inflammatory Responses to Gram-Negative Bacteria

Finja C. Hansen, Ann-Charlotte Strömdahl, Matthias Mörgelin, Artur Schmidtchen and Mariena J. A. van der Plas*

*** Correspondence:** Mariena J.A. van der Plas: mariena.van_der_plas@med.lu.se

Supplemental Videos. Phagocytosis of *E. coli* BioParticles in the presence of T-GKY25. Live cell imaging showing phagocytosis of *E. coli* BioParticles (green) in presence of T-GKY25 (red) by RAW 264.7 cells. The cells were cultured in an environmental chamber at 37 °C with 5% CO_2 and images were captured at 3 min intervals for 2 h. **Video 1** shows an overview of the phagocytosis of *E. coli* BioParticles in the presence of T-GKY25 by RAW 264.7 cells and **video 2** demonstrates a 3D projection of one selected macrophage. Videos are from a representative experiment (n=3).

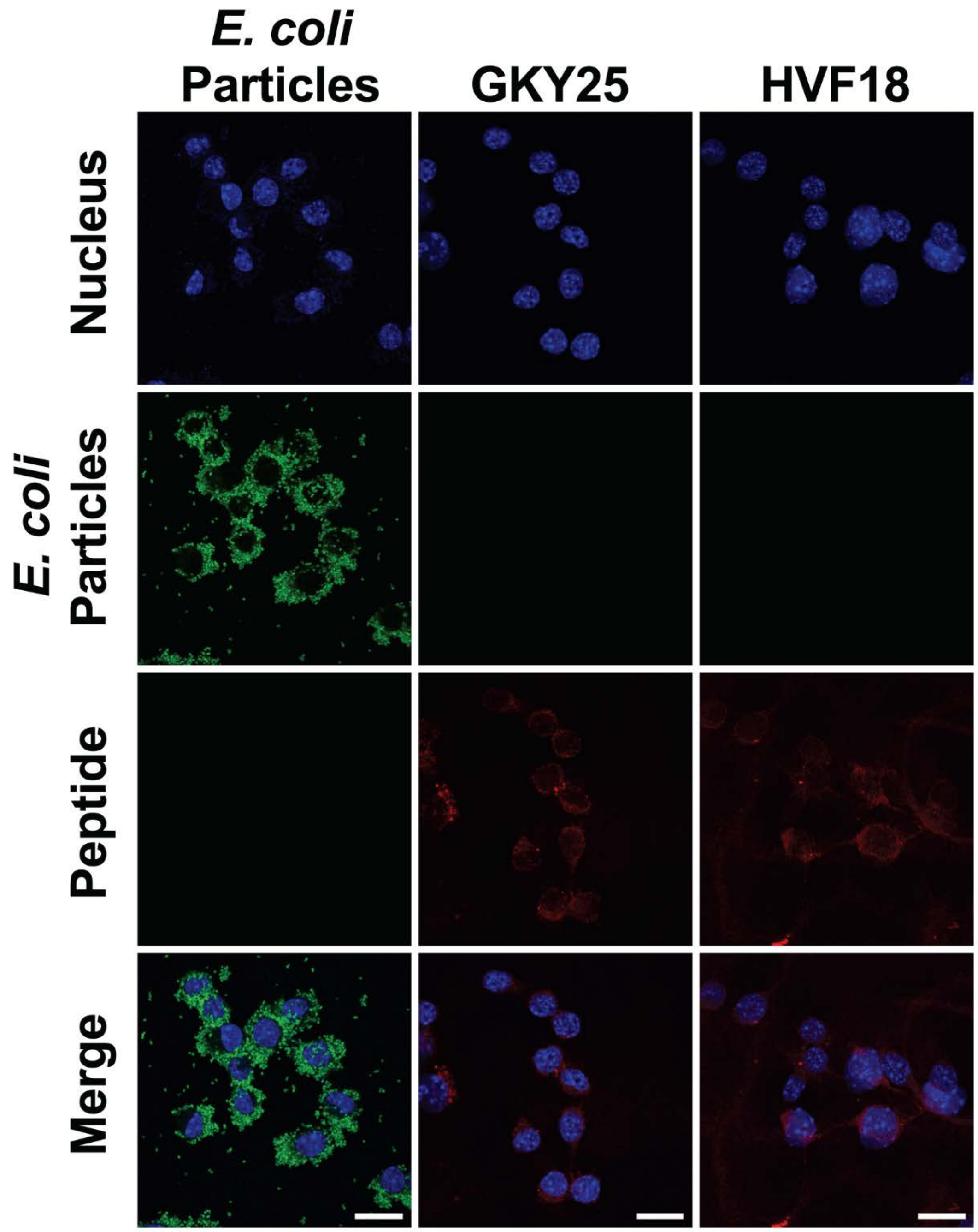

Supplemental Figure 1. Uptake of *E. coli* BioParticles or peptides in RAW 264.7 cells. RAW 264.7 cells were treated with either *E. coli* BioParticles (left panel), 2 μM GKY25 (middle panel) or 20 μM HVF18 (right panel) for 1.5 h at 37 °C. Cells were fixed, stained and analyzed by confocal microscopy using Zeiss LSM software. Nucleus DNA was stained with DAPI (blue), *E. coli* BioParticles (green) and TCPs were labeled with VFR17 antibody and Alexa 568 (red).

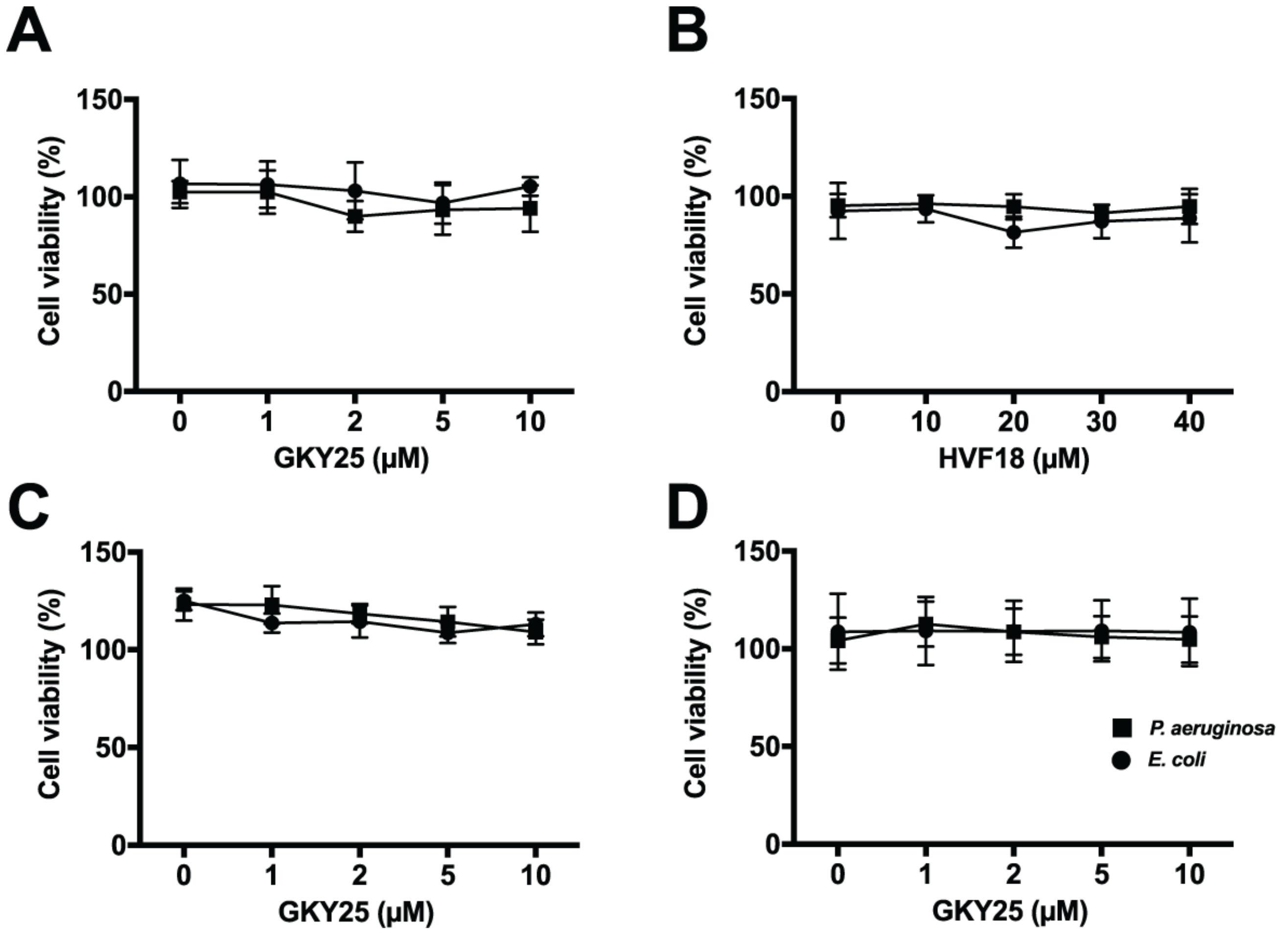

Supplemental Figure 2. Cell viability after stimulation with heat-killed bacteria. Cell viability was measured using a MTT assay after RAW Blue cells were treated for 20 h with heat-killed *Escherichia coli* ($4x10^5$ CFU/ml) or *Pseudomonas aeruginosa* ($4x10^6$ CFU/ml) in the presence of GKY25 **(A)** or HVF18 **(B**; n=3). **(C)** Heat-killed *E. coli* ($4x10^5$ CFU/ml) or *P. aeruginosa* ($4x10^6$ CFU/ml) were pre-incubated with GKY25 for 2 h, washed and added to RAW Blue cells. After 20 h, cell viability was measured (n=4). **(D)** Cell viability of stimulated RAW 264.7 cells with heat-killed *E. coli* ($4x10^5$ CFU/ml) or *P. aeruginosa* ($4x10^6$ CFU/ml) in the presence of GKY25 (n=5; means ± SEM).
